# Case report: a case of multiple splenic abscesses in a child and literature review

**DOI:** 10.3389/fped.2023.1162527

**Published:** 2023-05-05

**Authors:** Yingchun Yang, Jian Liu, Zebing Zheng, Chengyan Tang, Daiwei Zhu, Xingrong Xia, Lu Huang, Qing Du, Yongxing Hao, Yuanmei Liu, Zhu Jin

**Affiliations:** ^1^ Department of Pediatric Surgery, Affiliated Hospital of Zunyi Medical University, Zunyi, China; ^2^ Department of Pediatric Surgery, Guizhou Children Hospital, Zunyi, China

**Keywords:** splenic abscess, multiple splenic abscesses, children, laparoscopic total splenectomy, enhanced magnetic resonance imaging

## Abstract

Splenic abscesses in children are very rare, and multiple splenic abscesses are rarer. These lesions are difficult to diagnose quickly because of their low incidence and the low specificity of the associated clinical and imaging findings. The treatment of splenic abscesses includes conservative treatment, percutaneous drainage, and splenectomy, but the selection criteria for treatment are still unclear. We present a case of a 13-year-old girl with multiple splenic abscesses. Her blood culture report was negative. We eventually confirmed the diagnosis by enhanced magnetic resonance imaging (MRI). The patient underwent a successful laparoscopic total splenectomy, and her symptoms were resolved thereafter.

## Introduction

Splenic abscesses are rare but potentially fatal condition. This disease is more common in adults than in children (1) and is mostly unifocal. The most common causative organisms are Enterobacteriaceae, gram-positive cocci, and anaerobic bacteria (2–4). The predominant symptoms are fever, left upper quadrant abdominal pain, nausea and vomiting (5). Herein, we report a case of multiple splenic abscesses in a child and summarize the clinical characteristics, diagnosis and treatment of this disease.

## Case

**Figure 1:**
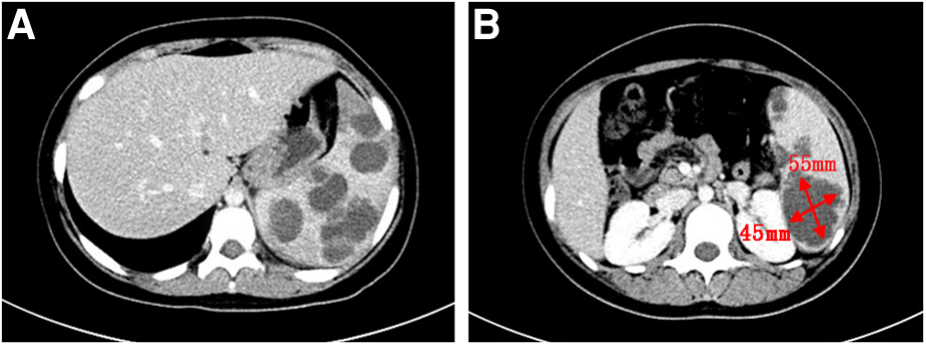
Contrast-enhanced abdominal CT scan. (**A**): Multiple low-density lesions of the spleen. (**B**): The largest lesion measures 45 × 55 mm.

**Figure 2:**
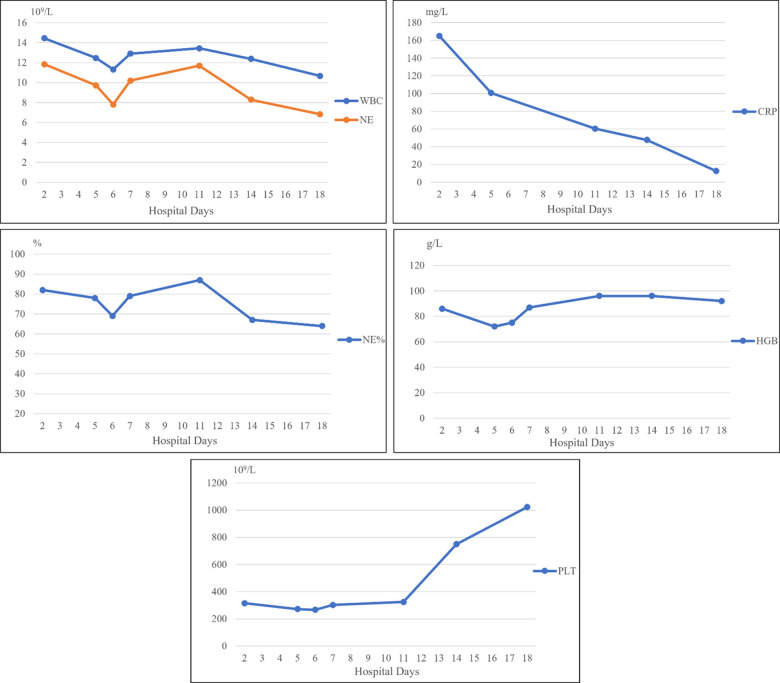
WBC, NE and NE% decreased after antibiotic treatment, increased slightly on the first postoperative day, and then continued to decline to the normal values. CRP continued to decrease after various interventions. These trends demonstrate that our treatment was effective. HGB continued to decline on admission, rebounded after we administered a blood transfusion, and was slightly low after surgery.

**Figure 3:**
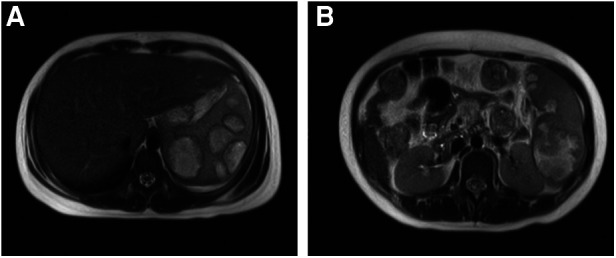
Contrast-enhanced MRI. (**A**): Splenomegaly with up to 7 abscesses in the same axial plane. (**B**): The largest abscesses.

**Figure 4:**
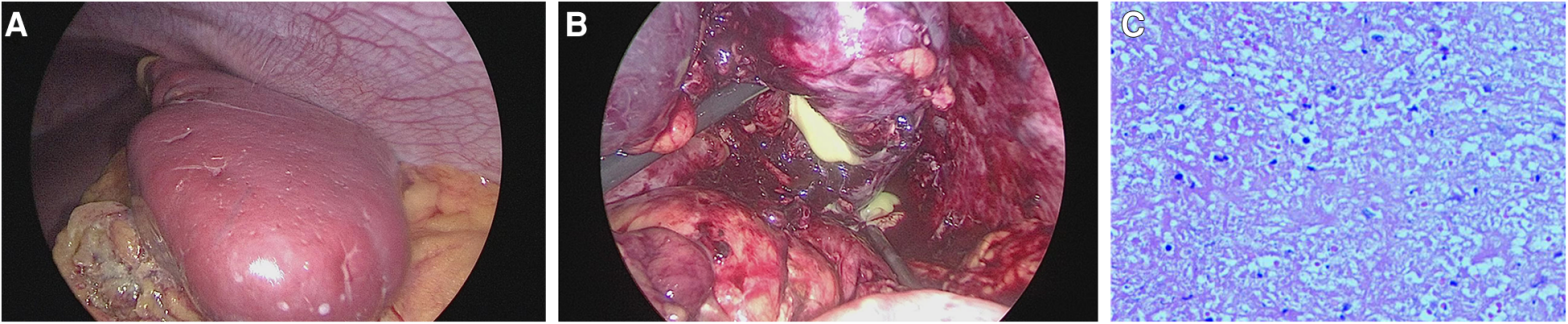
Intraoperative images and histopathology. (**A**): The spleen was enlarged (15 × 12 × 8 cm), and its surface was uneven and partially white. (**B**): Pus coming out of the spleen. (**C**): Chronic suppurative splenic inflammation with focal necrosis, abscess formation, local histiocytic hyperplasia, blood sinus dilatation and hemorrhage, and interstitial fibrous tissue hyperplasia.

A 13-year-old girl presented with persistent dull pain in the left upper abdomen one week before admission; the pain worsened 1 day prior to admission, accompanied by vomiting. Fever was present during this period, but the specific temperature was unknown. No special treatment was given. The physical examination showed only tenderness in the left upper abdomen and no special conditions in other important organs and systems. Gastrointestinal ultrasonography indicated splenomegaly with multiple dark fluid-filled areas and pelvic effusion. Abdominal contrast-enhanced computed tomography (CT) indicated multiple low-density lesions of the spleen (Figure 1A). The enhanced scan showed annular enhancement, with the largest being 45 × 55 mm (Figure 1B). Vascular tumors or other lesions were considered. Splenomegaly and a small amount of pelvic effusion were also observed. Bone marrow aspiration on the second day after admission showed that the proportion of neutrophils increased with increasing NAP score and decreasing erythron series. Laboratory examination: white blood cell count (WBC) 14.45 × 10^9^/L, neutrophil ratio (NE%) 82%, neutrophil (NE) 11.85 × 10^9^/L, hypersensitive C-reactive protein (CRP) 165.086 mg/L, hemoglobin (HGB) 86 g/L. Female tumor-related antigen examination indicated carbohydrate antigen-125 112 and ferritin 477. After 3 days of cefminox treatment, WBC, NE%, NE and CRP decreased (Figure 2). Since the patient's HGB was low at 72 g/L, 1 unit of WBC-free erythrocyte suspension was given. MRI of the upper abdomen revealed splenomegaly with multiple abscesses and slight perisplenic exudation (Figures 3A,B). Because (i) MRI clearly indicated multiple splenic abscesses, (ii) the anti-inflammatory effect of cefminox was not obvious, and (iii) a fever spike occurred at night (39.1°C), we replaced the antibiotic with cefoperazone sodium and sulbactam sodium and planned to perform surgical treatment as soon as possible. The next day, 6 days after admission, the blood bacterial culture was negative. After HGB rose to 75 g/L, another unit of WBC-free erythrocyte suspension was given. On the 7th day, laboratory examination showed that WBC, NE% and NE had increased slightly, failing to reflect any obvious controlling effect of antibiotics. Based on the results of all examinations, laparoscopic total splenectomy was performed on the 8th day for radical treatment. Splenomegaly (15 × 12 × 8 cm) was observed during the operation. The surface of the spleen was uneven and partially white (Figure 4A), and the spleen was closely adhered to the greater omentum and liver, with edema. After the spleen was completely resected, the splenic tissue was placed into a specimen bag, cut up and removed, and bacterial culture was performed. No bacterial growth was observed. Postoperative pathological findings included chronic suppurative splenic inflammation with focal necrosis, abscess formation, local histiocytic hyperplasia, blood sinus dilatation and hemorrhage, and interstitial fibrous tissue hyperplasia (Figure 4C). The CRP decreased significantly on the first day after surgery. The platelet count (PLT) had been in the normal range before operation and increased rapidly after splenectomy. We believe that this is mainly due to diminished phagocytosis of aged cells after splenectomy. The patient's immunoglobulin M (IgM) level was 0.3 g/L before surgery and decreased to 0.26 g/L after surgery. The levels of the other immunoglobulins remained normal, as did the levels of complement C3 and C4. On the 13th day after admission, the bacterial culture of peritoneal drainage was negative. Hydrothorax ultrasound indicated bilateral pleural effusion. On the 14th day, inflammatory indicators began to recover. On the 18th day, WBC, NE%, NE and CRP completely returned to normal. Epstein‒Barr virus DNA and cytomegalovirus DNA were negative. The pleural effusion was well absorbed, although an ultrasound of pleural effusion in the chest and abdomen indicated some remaining pleural effusion on the left. On the 19th day, a reexamination of the upper abdominal enhanced CT revealed a small amount of fluid/blood in the operative area after splenectomy. The patient was discharged on day 21. We followed the patient up for three months after the operation. The patient was well, with a normal blood routine test and no recurrence of the abscesses.

## Discussion

Splenic abscesses are very rare; a series of autopsy studies have found that their prevalence is 0.2%–0.7% (6, 7). We reviewed some cases of splenic abscesses in children. Clinical, laboratory, imaging, treatment and etiology variables in splenic abscesses in children are summarized (Table 1). Due to the physiological function of the spleen against infection, splenic abscesses are usually associated with other diseases or immunocompromised states, such as splenic trauma, malignant tumors, diabetes, or systemic infection (3). Immunodeficiency is also the main pathogenic factor of splenic abscesses (16). With the increasing incidence of malignant tumors, organ transplantation, immunosuppressive therapy, and AIDS, the risk of splenic abscesses is also gradually increasing (3, 17). However, as these underlying diseases are much less common than in adults, splenic abscesses are rare in children. The onset age of splenic abscesses in children is usually over 10 years old. The most common causes of splenic abscesses in children include secondary infections transmitted through the blood, hematological abnormalities, typhoid fever, and tuberculosis, among others. Infective endocarditis, otitis media and appendicitis are common causes of secondary infections. Among the causative hematological abnormalities, the most common is leukemia, followed by aplastic anemia and anemia of other etiologies (8, 11).

**Table 1:** Clinical, laboratory, imaging, treatment and etiology variables associated with splenic abscesses in children.

Variables	Subhasis et al. 2000–2008 (8)	Ahmad et al. 1990–2010 (9)	Kamal et al. 2009 (10)	Claudia et al. 1907–1989 (11)	Other individual cases 2012–2017 (12–15)
Number of cases	18	17	3	56	4
Males:females	15:3	14:3	3:0	30:18	2:2
Mean age (range)	9 (3–17) y	8 (5–14) y	10 (9–12) y	(0–18) y	14 (12–15) y
**Clinical presentation**					
Fever	18 (100%)	15 (88%)	3 (100%)	51 (91%)	4 (100%)
Anorexia	18 (100%)	Not described	Not described	8 (14%)	Not described
Splenomegaly	12 (67%)	12 (70%)	3 (100%)	26 (46%)	2 (50%)
**Laboratory tests**					
Leukocytosis	9 (50%)	12 (70%)	3 (100%)	Not described	3 (75%)
Thrombocytosis	12 (67%)	10 (58%)	Not described	Not described	Not described
Microbial culture(+)	3 (17%)	14 (82%)	2 (67%)	30 (54%)	2 (50%)
Blood culture(+)	1 (8%)	8 (47%)	1 (33%)	17 (30%)	0 (0%)
G(−)	18 (100%)	Not described	1 (33%)	11 (20%)	3 (75%)
G(+)	Not described	Not described	2 (67%)	18 (32%)	0 (0%)
**Imaging**					
Ultrasound	18 (100%)	15 (88%)	3 (100%)	20 (36%)	2 (50%)
CT	18 (100%)	8 (47%)	2 (67%)	16 (29%)	2 (50%)
Multiple abscesses	9 (50%)	0 (0%)	1 (33%)	29 (52%)	2 (50%)
**Treatment**					
Antibiotics	18 (100%)	17 (100%)	3 (100%)	38 (68%)	4 (100%)
Drainage	10 (56%)	15 (88%)	2 (67%)	9 (16%)	3 (75%)
Splenectomy	0 (0%)	2 (12%)	1 (33%)	28 (50%)	0 (0%)
**Predisposing factors**					
Typhoid fever	9 (50%)	5 (29%)	Not found	Not found	Not found
Tuberculosis	1 (6%)	1 (6%)	Not found	Not found	Not found
Diabetes mellitus	Not found	1 (6%)	Not found	Not found	Not found
Distant infections	1 (6%)	Not found	2 (67%)	16 (30%)	Not found
Hematological abnormalities	1 (6%)	2 (12%)	Not found	26(46%)	Not found
Anatomic	Not found	Not found	Not found	6(11%)	Not found

*G(−): gram-negative bacteria; G(+): gram-positive bacteria.

Splenic abscesses have no specific clinical manifestations. Most patients usually seek treatment due to fever and left upper abdominal pain, and their hematological examinations will generally find increased WBC (2, 5, 8, 9, 11). Blood culture is of great significance in clarifying the etiology and diagnosis. It has been reported that 24%–80% of splenic abscesses test positive on blood culture, and multiple abscesses are more likely to be the result of bloodborne transmission, while other routes of transmission mostly cause independent abscesses (2). Aseptic splenic abscesses also exist and are often the first manifestation of inflammatory bowel disease, especially Crohn's disease, with gastrointestinal symptoms (18). Our patient showed no signs of digestive tract inflammation, such as diarrhea, rectal tenesmus or changes in stool characteristics.

Due to the low sensitivity and specificity of clinical symptoms and laboratory markers, imaging plays a crucial role in diagnostic examination (19). Digital x-ray imaging can assist in diagnosis. In some patients, left diaphragmatic elevation, left pleural effusion, and abnormal soft tissue masses in the left upper quadrant can be observed (20), but these signs lack specificity. The sensitivity of abdominal ultrasound for splenic abscesses are estimated to be 75%–93% (4, 19). Most abscesses are oval or round lesions with low or almost no echo, irregular walls, and mild to moderate acoustic enhancement at the distal end, which can be considered somewhat specific (17). Splenic abscesses can occur as a complication of infective endocarditis, which can aggravate the disease and increase mortality (21, 22). Davido et al. proposed that any splenic abscesses found must be examined by cardiac ultrasound to exclude the possibility of endocarditis (2). Infective endocarditis is often characterized by fever, leukocytosis and splenomegaly, a symptom profile resembling that of splenic abscesses, but infective endocarditis develops more rapidly and poses a greater risk. Therefore, we also believe that when splenic abscesses are suspected, echocardiography should be performed to rule out infective endocarditis. It has been reported that the sensitivity of CT for detecting splenic abscesses can reach 100%, with the lesions typically presenting as low-density masses with peripheral enhancement (17). After injection of a contrast agent, uneven enhancement of splenic tissue is observed, and then its parenchyma gradually becomes evenly enhanced (19). Compared with CT, MRI has superior contrast, clarity and multidirectional imaging capabilities. MRI has higher resolution than CT for the surrounding soft tissue and can better judge the situation of lesions invading adjacent tissue. MRI is superior to CT in displaying splenic microabscesses (23). In our case, the initial CT examination did not definitively identify the lesions as abscesses. It was the clear indication on MRI that allowed us to diagnose splenic abscesses conclusively. Ultrasound and MRI are extremely sensitive in the early diagnosis of splenic infection and can be considered suitable early diagnosis methods (19, 24).

It has been suggested in the literature that the etiology of splenic abscesses is unknown in approximately one-third of cases (25); these cases can be considered primary splenic abscesses. However, Michale et al. proposed that primary splenic abscesses do not completely lack specific clinical characteristics (26). The patients they studied were all in good physical condition before onset, with rapid onset, large abscesses, and negative bacterial cultures. No other diseases were found in our case. The patient had no previous surgical history or history of infection, and her physical fitness was at the same level as that of normal children of the same age. A week after the onset of the disease, imaging examination found that the volume of the abscesses had occupied almost the entire spleen, and there were up to seven abscesses at the same level in the scan. After admission, blood culture and virus tests were negative. No other gastrointestinal symptoms were found before or after the course of the disease except vomiting for one day; therefore, this case did not meet the criteria for aseptic splenic abscesses. According to the findings from CT and MRI, we diagnosed the patients with splenic abscesses, a diagnosis that was confirmed during the operation. This case has no clear etiology, which is consistent with the clinical characteristics of primary multiple splenic abscesses as summarized in the literature.

There is no gold standard for the treatment of splenic abscesses, and individual treatment should be tailored to each patient's condition. The general treatment plan is antibiotics first. If there is no obvious improvement, we then choose either abscess drainage or splenectomy according to the patient's condition. To maintain the immune function of the spleen, many experts believe that the optimal treatment for pediatric splenic abscesses is broad-spectrum intravenous antibiotics and early percutaneous drainage, especially for isolated splenic abscesses, which respond well to treatment and do not require splenectomy (8, 9, 27). Although the success rate of splenectomy has been reported to be 86%–94%, it carries risks of surgical drainage and post-splenectomy sepsis and does not preserve splenic tissue which these experts consider very important; on this basis plus the high success rate and low cost of drainage, they believe that percutaneous drainage should be the preferred treatment for splenic abscesses (3, 17, 28). It has been suggested that preservation of the spleen is especially valuable for children with immunocompromised and incomplete immune function (29), and percutaneous drainage should be considered the preferred treatment at this time. However, some experts believe that splenectomy has a better therapeutic effect than percutaneous drainage or antibiotic therapy alone and that intravenous antibacterial therapy combined with splenectomy should be the first choice for the treatment of splenic abscesses (4, 16, 17). When percutaneous drainage fails due to multiple abscesses, tight adhesion around the spleen, or other reasons, a splenectomy or open drainage is needed. Follow-ups from medical centers showed that no patients had surgical complications or sequelae related to their spleen lesions (16). Although there was a slight decrease in our patient's inflammatory indicators after initial antibiotic treatment, the change was not significant, and there was no sign of reduced absorption in the abscesses. There were too many abscesses in our patient, occupying too large a volume in the spleen. Without timely intervention and treatment, there would have been a considerable risk of abscess rupture, leading to aggravation of disease and even death. According to the imaging findings, there was little normal splenic tissue left, and even if puncture drainage were performed, the effect would not be substantially different from that of splenectomy. There was also the risk of incomplete drainage of the abscesses, and the child was 13 years old, with mostly mature immune function; therefore, we directly performed a laparoscopic total splenectomy. After surgery, the child's inflammatory markers quickly returned to normal. No other symptoms appeared, and the patient made a good recovery. She was followed up for three months after the operation, and no physical discomfort was found. A routine blood test and ultrasound showed no obvious abnormalities.

## Conclusion

Splenic abscesses in children are rare, and the associated clinical manifestations, laboratory test results and imaging findings are not highly specific. The possibility of splenic abscesses should be considered in the presence of unexplained fever, left upper abdominal pain, palpable abdominal mass, and changes in the imaging features of the spleen. CT may be preferred as a diagnostic aid, as it currently is at many centers, but in cases in which CT is inconclusive, our experience suggests that the addition of MRI is warranted. Blood culture should be carried out as early as possible, and sensitive antibiotics should be taken to block the development of the disease in time. Percutaneous drainage can be considered for a single large splenic abscess, and splenectomy can be considered for older children as well as children with multiple splenic abscesses.

WBC, NE and NE% decreased after antibiotic treatment, increased slightly on the first postoperative day, and then continued to decline to the normal values. CRP continued to decrease after various interventions. These trends demonstrate that our treatment was effective. HGB continued to decline on admission, rebounded after we administered a blood transfusion, and was slightly low after surgery.

## Data Availability

The original contributions presented in the study are included in the article, further inquiries can be directed to the corresponding author/s.
